# Löfgren Syndrome: Clinical Presentation, Clinical Course, and Literature Review

**DOI:** 10.7759/cureus.33651

**Published:** 2023-01-11

**Authors:** Rui Flores, Sofia Caridade

**Affiliations:** 1 Cardiology, Hospital de Braga, Braga, PRT; 2 Internal Medicine, Hospital de Braga, Braga, PRT

**Keywords:** rare case, erythema nodosum, anemia, löfgren syndrome, acute sarcoidosis

## Abstract

Löfgren syndrome is an acute presentation of sarcoidosis that comprises fever, bilateral and symmetric hilar lymphadenopathies, polyarthritis, and erythema.

We present the case of a 34-year-old male patient who presented with ankle monoarthritis without a history of a traumatic event. Contralateral ankle arthritis associated with erythema nodosum and fever developed one week later. Laboratory tests showed anemia, thrombocytosis, and elevated inflammatory parameters. A chest CT revealed symmetrical mediastinal and hilar adenopathies. A transbronchial biopsy was compatible with granulomatous lymphadenitis, and the diagnosis of Löfgren syndrome was confirmed.

Our case report and literature review emphasize the wide web of mimicry of acute sarcoidosis. Secondary forms of acute sarcoidosis are likely to benefit from additional and more complex immunomodulatory therapies. Close monitoring and follow-up should be conducted because it is possible that these patients experience higher rates of recurrence or relapse.

## Introduction

Löfgren syndrome (LöS), an acute variant of sarcoidosis, was initially described by Sven Löfgren in 1953 after the compilation of more than 100 cases of febrile young adults showing a combination of erythema nodosum, ankle periarthritis, and bilateral hilar lymphadenopathies [1,2]. The overwhelming majority of patients with LöS recover spontaneously and completely, although some cases require the use of steroids and immunosuppressors to achieve remission [3]. Despite being described for more than 60 years, there is still a lack of predictors that can allow us to infer the need for therapy in these patients.

Here, we report the case of an idiopathic LöS with hepatic and hematologic involvement and perform a review of case reports and case series published in the literature. Our aim is to revise the potential predictors of prognosis and features that can indicate the need for early immunotherapy. We conducted a search on PubMed using the following terms: “Löfgren syndrome” OR “acute sarcoidosis” OR “acute sarcoid arthritis.” We filtered for case reports/series and English-language papers. After a thorough reading of abstracts, we excluded cases in which the diagnosis of LöS was unclear and full-text papers that were unobtainable.

## Case presentation

A 34-year-old male patient, a native and resident of Portugal, presented to the Emergency Department (ED) for edema and pain in his right ankle. He worked as a locksmith and had a medical history of herniated lumbar disc and hemorrhoidal disease. No chronic medication use was reported, and no previous allergies were known. He denied a history of local trauma. Bone fractures were excluded with a foot and ankle radiography, and he was discharged with a non-steroidal anti-inflammatory drug (NSAID).

The patient returned about one week later to the ED complaining of contralateral ankle edema and pain. He also reported fever, dry cough, and a painful rash on the lower extremities associated with pruritus. Additionally, he mentioned a significant weight loss in the past few weeks. A review of the remaining systems, allergies, and family history was unremarkable.

On physical examination, he showed signs of pitting edema, tenderness, and erythema at the right ankle, as well as bilateral, erythematous, tender, and rounded subcutaneous nodules in the upper and lower limbs of a reddish color. Physical examination was otherwise normal.

Laboratory tests at admission revealed a hemoglobin level of 11.8 g/dL (normal range (NR) = 13.5-17.5 g/dL), mean corpuscular volume of 80.3 fL (NR = 81.895.5 fL), white blood cell (WBC) count of 13,000/µL (NR = 4,000-11,000/µL) with 83.2% neutrophils, platelet count of 582 × 10^3^/µL (NR = 150-450 × 10^3^/µL), C-reactive protein (CRP) of 162 mg/L (NR = <3 mg/L), albumin of 2.6 g/dL (NR = 3.4-5 g/dL), and lactic dehydrogenase (LDH) of 268 IU/L (NR = 87-124 IU/L). All main laboratory findings are presented in Table 1.

**Table 1:** Laboratory tests. MCV = mean corpuscular volume; RDW = red cell distribution width; WBC = white blood cells; AST = aspartate aminotransferase; ALT = alanine aminotransferase; LDH = lactic dehydrogenase; GGT = gamma-glutamyl transferase; INR = international normalized ratio; CRP = C-reactive protein; ESR = erythrocyte sedimentation rate; ANA = antinuclear antibodies; ENA = extractable nuclear antigen; dsDNAab = double-stranded DNA antibodies; ANCA = antineutrophil cytoplasmic antibodies; ACE = angiotensin-converting enzyme; ASO = antistreptolysin O; TSH = thyroid-stimulating hormone

Laboratory parameter		Admission	Hospitalization 1 day later	Hospitalization 5 days later	Discharge 10 days later	Outpatient 3 months later	Normal range
Hemoglobin (g/dL)		11.8	11.0	11.1	13.1	14.1	13.5–17.5
MCV (fL)		80.3	80.2	79.5	79.1	87.5	81.8–95.5
RDW (%)		14.2	14.1	14.1	14.0	13.9	11.6–14.0
WBC count (/µL)		12 900	11 000	9 200	6 500	6 800	4,000–11,000
	Neutrophils (%)	83.2	78.4	68.2	60.0	60.4	-
	Eosinophils (%)	1.2	2.6	3.9	4.1	4.3	-
	Basophils (%)	0.3	0.2	0.4	0.6	0.4	-
	Lymphocytes (%)	10.4	13.6	21.9	29.8	28.6	-
	Monocytes (%)	4.7%	5.2	5.6	5.5	6.3	-
Platelet count (/µL)		582,000	503,000	607,000	287,000	293,000	150,000–450,000
Corrected serum calcium (mEq/L)		-	9.4	-	-	-	8–10
Calcium in a 24-hour urine sample (mg)		-	220	-	-	-	<300
Albumin (g/dL)		2.6	-	-	-	3.3	3.4–5.0
Total proteins (g/dL)		-	7.3	-	-	6.9	6.4–8.2
AST (IU/L)		9	13	13	15	25	15–37
ALT (IU/L)		24	22	21	30	23	12–78
LDH (IU/L)		268	167	164	153	206	87–124
Total bilirubin (mg/dL)		0.41	0.40	0.45	0.54	0.56	0.1–1.0
Alkaline phosphatase (IU/L)		224	116	97	82		45–117
GGT (IU/L)			90	68	54	34	15–85
Prothrombin time (seconds)		14.4	-	-	-	13.0	8.0–14.0
INR		1.21	-	-	-	1.1	0.8–1.2
CRP (mg/L)		162	136	64	8.8	14	<3
ESR (mm/hour)		-	98	-	23	10	1–15
ANA		-	1/160	-	1/80	-	-
ENA		-	Negative	-	-	-	-
dsDNAab (IU/mL)		-	2.5	-	-	-	<30
C3 (mg/dL)		-	117	-	-	-	90–180
C4 (mg/dL)		-	25	-	-	-	10–40
C_H_50 (IU/mL)		-	61	-	-	-	23–60
ANCA		-	Negative	-	-	-	
Rheumatoid factor		-	Negative	-	-	-	
ACE (U/L)		-	34	-	-	-	8–55
ASO (IU/mL)		-	291	-	-	-	0–408
Immunoglobin A (mg/dL)		-	412	-	294	-	70–400
Immunoglobin G (mg/dL)		-	1720	-	1400	-	700–1,600
Immunoglobin M (mg/dL)		-	63.7	-	68.9	-	40–230
TSH (uIU/mL)		-	2.840	-	-	-	0.358–3.740

**Figure 1:**
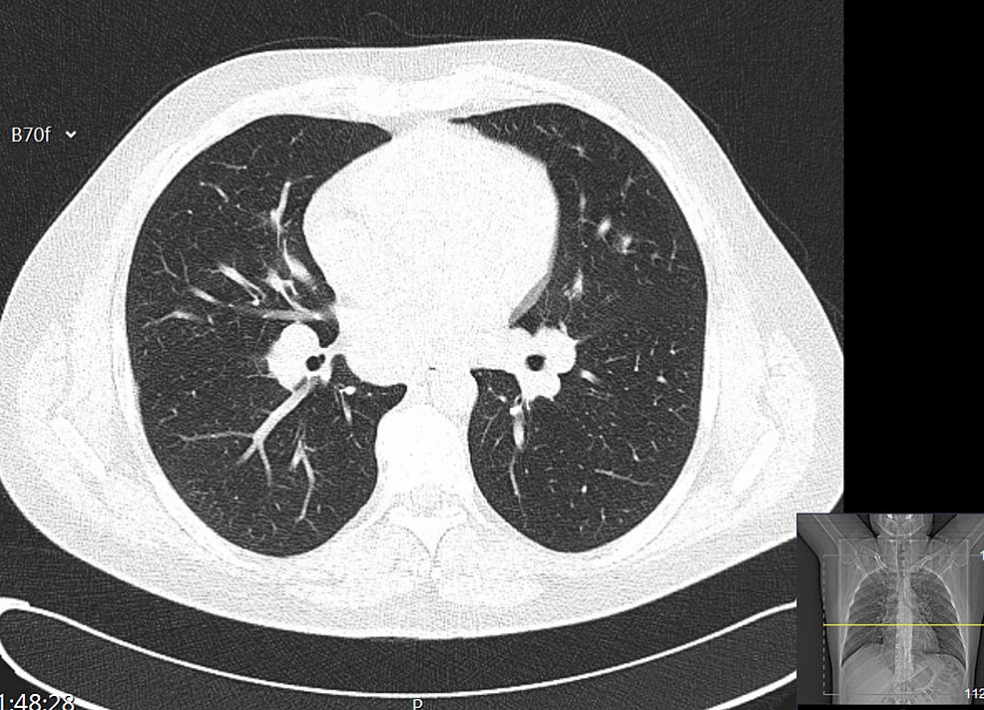
CT thoracic scan.

A thoracic radiography showed symmetric perihilar adenomegalies. The lungs were clear. A CT scan of the chest showed multiple symmetrical mediastinal and hilar adenopathies, alongside a few micronodules in the lung parenchyma (Figure 1).

An abdominal ultrasound showed moderate hepatomegaly (longitudinal diameter of 17.7 cm in the mid-clavicular line), but bile ducts were normal. Ultimately, the patient was admitted to the Internal Medicine Department for an investigation of fever, skin lesions, mediastinal lymphadenopathies, and migratory ankle arthritis.

During hospitalization, further laboratory tests showed a striking increase in the erythrocyte sedimentation rate (ESR) (98 mm/hour, NR = 0-22 mm/hour). The autoimmunity panel, including DNA double-stranded antibodies (dsDNAab), complement level, antineutrophil cytoplasm antibodies (ANCA), anticardiolipin antibodies, lupus anticoagulant, anti-β2-glycoprotein antibodies, rheumatoid factor, and angiotensin-converting enzyme (ACE) levels, was unremarkable. The first antinuclear antibodies (ANA) titers were 1/160, but extractable nuclear antigen (ENA) antibodies were negative. Normal ANA titers were posteriorly found. Infectious serologies for syphilis, Epstein-Barr virus, hepatitis B and C, and human immunodeficiency virus were negative. The intradermal tuberculin (Mantoux) test was anergic, and the interferon-gamma release assay (IGRA) was negative. Antistreptococcal O (ASO) titers and nasopharynx swab tests were also unremarkable. Serum and urinary calcium were normal. Iron kinetics, folate, and vitamin B12 levels were also normal, as were the serum protein electrophoresis, protein immunoelectrophoresis, and level and ratio of serum and urinary light chains. A skin biopsy of the lower limbs revealed signs of panniculitis compatible with erythema nodosum.

An echoendoscopy-guided bronchoscopy was conducted to perform an aspiration transbronchial biopsy. Histologic examination confirmed granulomatous lymphadenitis, which was compatible with the diagnosis of acute sarcoidosis. Bronchoalveolar lavage (BAL) was normal, except for a high CD4+/CD8+ ratio and lymphocyte count.

The diagnosis of LöS was formulated. The patient had a favorable clinical and analytical evolution during a 10-day hospitalization. Glucocorticoids were not started, and discharge was given after symptoms became mild.

The follow-up visit was arranged for three months after discharge. The patient had complete resolution of skin lesions, respiratory symptoms, and arthritis. Laboratory tests were inapparent, and chest CT showed resolution of mediastinal lymphadenopathies.

## Discussion

Sarcoidosis is an immune-mediated and complex multiorgan disorder [3,4]. Despite its worldwide distribution, certain ethnicities and age groups are more affected, reflecting genetic susceptibility and common environmental factors [4,5]. Most studies support the role of several external agents in triggering an inflammatory and granulomatous response of the host [3]. Despite many years of research, its mechanisms remain poorly understood [4].

The clinical course of sarcoidosis is variable and sometimes unpredictable, ranging from acute and self-limited to chronic, progressive, and debilitating [5]. The most commonly affected organs are the lungs, although a significant proportion of patients show extrapulmonary involvement, including skin, lymph nodes, and eyes [3,4,6].

LöS is an acute presentation of sarcoidosis that comprises a pentalogy of clinical manifestations, including bilateral and symmetric hilar lymphadenopathies, fever, polyarthritis, erythema nodosum, and uveitis, and is often associated with good prognosis [3,6,7]. The combination of all five clinical features has a high specificity for the diagnosis of LöS, obviating the need for a biopsy for a definitive diagnosis [7]. Furthermore, non-classical or atypical presentations require histological confirmation of granulomatous disease and the inherent differential diagnosis, such as infections or autoimmune diseases [7,8]. Despite its resemblance to neoplastic or paraneoplastic syndromes, most cases are self-limited and do not require specific therapy [3]. Treatment with NSAIDs is the first line for symptomatic patients, but some patients have refractory or persistent manifestations that require glucocorticoids or other immunomodulators [9]. About 10% of cases progress to chronic disease extending longer than two years despite immunosuppressive therapy [10-12].

We report a case of acute sarcoidosis with atypical involvement, along with the classical symptoms and signs of LöS. Although rarely described, liver and hematological involvement seem natural in a pro-inflammatory and immune systemic condition. Nevertheless, the patient recovered completely in a few months, which led us to conclude that features such as anemia, thrombocytosis, and hypoalbuminemia do not seem to modify the good prognosis of LöS.

A review of case reports is presented in Table 2 with the description of 22 patients who presented with LöS.

**Table 2:** A list of case reports. ACE = angiotensin-converting enzyme; CRP = C-reactive protein; ESR = erythrocyte sedimentation rate; FMF = familial Mediterranean fever; NS = not specified; od = once daily; sc = subcutaneous; F = female; M = male

Reference	Age	Sex	Trigger	ESR (mm/hour)	CRP (mg/L)	ACE (UI/L)	Treatment	Organs involved	Outcome
von Knorring and Selroos (1976) [13]	71	F	-	75–99	NS	NS	NSAIDs, multiple courses of steroids	Lymph nodes, skin, joints	Rheumatic polymyalgia was soon diagnosed, and a chronic course of low-dose steroids was initiated, leading to full recovery of acute sarcoidosis
Halevy et al. (1980) [14]	16	M	-	96	NS	NS	Prednisone 40 mg od, posteriorly weaned off	Lymph nodes, skin, joints	Full recovery after four months
Hillerdal et al. (1984) [15]	31	F	-	97	-	-	Prednisolone 60 mg od	Lymph nodes, skin, joints, lungs	After the initial favorable response, the patient eventually died from pulmonary aspergillosis
Johnston et al. (1984) [16]	41	M	-	41–80	NS	NS	Symptomatic treatment in the first episode, steroids (prednisolone 20 mg od) in the second episode	Lymph nodes, skin, joints, eyes	Recurrence in one month after spontaneous remission, full recovery after steroids
Iino et al. (1991) [17]	23	F	-	NS	103	29.2	NSAIDs	Lymph nodes, skin, joints	Full and spontaneous recovery
Palestro et al. (1992) [18]	32	F	-	NS	NS	NS	NSAIDs, steroids	Lymph nodes, skin, joints, eyes, liver, lungs	Improvement with steroids
Zabawski et al. (1997) [9]	26	M	Prior surgery (?)	69	94 (NR = <10)	NS	Prednisone due to failure to NSAIDs	Lymph nodes, skin, joints	Full recovery
Teh et al. (2000) [19]	30	M	-	50	NS	Normal	NSAIDs, prednisolone 30 mg od subsequently tapered for the following 12 months	Lymph nodes, skin, joints	Full recovery without recrudescence in 24 months
Stuveling et al. (2001) [20]	43	F	-	120	NS	Normal	NSAIDs	Lymph nodes, skin, joints, eyes	Full recovery
Ohta et al. (2006) [21]	26	F	-	43	7	23.6 (NR = 7.7–29.4)	Prednisolone 30 mg od, followed by weaning after the failure of NSAIDs	Lymph nodes, skin, joints, lungs	Full recovery and withdrawal of steroids after 18 months. No recurrence after six months
Patel et al. (2007) [22]	29	M	-	NS	NS	NS	NS	Lymph nodes, skin, joints, eyes, lungs, heart	NS
Bourdillon et al. (2007) [10]	36	F	-	60	80	89 (NR = 12–68)	Symptomatic	Lymph nodes, skin, joints, liver, peritoneum	Full recovery after six months
Marcoval et al. (2008) [23]	49	F	Desensitization injections for extrinsic asthma	NS	NS	57 (NR = 7–52)	Symptomatic	Lymph nodes, skin, joints	Spontaneous remission in two months
Dadban et al. (2009) [24]	55	F	-	NS	100 (NR = <10)	Normal	Oral colchicine and NSAIDs	Lymph nodes, skin, joints, eyes	Lost to follow-up
Erten et al. (2012) [25]	61	F	FMF (heterozygous mutation for E148Q)	124	215	63.2 (NR = 8–52)	Glucocorticoids (methylprednisolone 16 mg od), methotrexate 10 mg/week, posteriorly added colchicine after FMF diagnosis	Lymph nodes, skin, joints, lungs	Recovery after colchicine initiation
Klevtsova et al. (2015) [26]	27	F	-	Elevated	Elevated	NS	High-dose steroids	Lymph nodes, skin, joints, lungs	Full recovery in a few days
Kim et al. (2016) [27]	52	M	Therapy with nivolumab and ipilimumab	70–90 (NR = <20)	NS	Normal	Hydroxychloroquine 200 mg twice daily and high-dose steroids (IV methylprednisolone 1 mg/kg twice daily)	Lymph nodes, skin, joints	Recovered from LöS but eventually succumbed to malignancy
Rezgui et al. (2016) [28]	57	F	Systemic lupus erythematosus and amyloidosis	123	190	NS	Colchicine 1 mg od and chloroquine 200 mg od	Lymph nodes, skin, joints, liver, spleen, eyes, lungs, pericardium	NS
Mirzaei et al. (2017) [29]	47	F	Eyebrows tattooing	51 (NR = <29)	48 (NR = <10)	73 (NR = <40)	Steroids (multiple dosages, oral and intralesional), azathioprine (100 mg daily, suspended for hepatotoxicity), methotrexate 15 mg weekly, adalimumab 40 mg sc every two weeks along with 10 mg prednisolone	Lymph nodes; skin; joints; lungs	Recovered after a combination of adalimumab and steroids. Due to the re-emergence of symptoms after increasing the intervals of adalimumab, the patient maintained adalimumab after nine months of follow-up
Saltman et al. (2017) [30]	61	F	-	95	Elevated	NS	Oral prednisone 50 mg od due to failure to non-steroidal anti-inflammatory drugs	Lymph nodes, skin, joints	Full recovery
Graf et al. (2018) [31]	44	F	Treatment with alemtuzumab	NS	NS	77 (NR = 20–70)	Oral steroids.	Lymph nodes, joints	Remission
Kronbichler et al. (2018) [32]	46	F	Treatment with rituximab	NS	NS	Slightly elevated	Low-dose steroids (methylprednisolone 16 mg daily initially, followed by weaning)	Lymph nodes, skin, joints	Full recovery

Most patients are young females with a mean age of 41 years (ranging from 16 to 71 years).

Although missing in several reports, a significant proportion of patients had normal or slightly elevated ACE levels, which corroborates current literature focusing on the controversial role of ACE in the diagnosis of sarcoidosis [33,34]. This can be due to a hypothetical correlation between ACE levels and the severity of the disease as LöS constitutes a supposedly benign variant of acute sarcoidosis. However, ACE levels are independent of the pulmonary prognosis of systemic sarcoidosis and seem unable to predict the overall prognosis of LöS. High ACE levels are compatible with a full recovery, and normal ACE levels may occur in patients in need of treatment, such as steroids or other immunosuppressive drugs. Additionally, high ACE levels lack specificity as they can be associated with systemic storage diseases, liver diseases, diabetes mellitus, other granulomatous or autoimmune diseases, and infections [33,35].

Bilateral hilar lymphadenopathies, erythema nodosum, and arthralgias are the most frequently described features of LöS in the literature. Uveal disorders are also labeled as one of the cornerstones for diagnosis [3,4,6]. Nevertheless, uveitis is rarely described. It is unknown whether this represents an underdiagnosis of subclinical disease or a less common association. Lung nodules or infiltrates, as well as cardiac, peritoneal, or liver involvement, are rarely described.

Full recovery was a common denominator in all but two cases, in which both patients died of conditions unrelated to the progression of acute sarcoidosis. One case of pulmonary aspergillosis secondary to steroidal use was described by Hillerdal et al. in 1984 [15], and another case of LöS associated with the use of biologic immunomodulators for a metastatic urothelial carcinoma was described by Kim et al. in 2016 [27]. In the latter, the patient eventually succumbed to the primary tumor. Still, 17 out of the 22 patients required at least one course of steroids or other immunosuppressive drugs to achieve remission. The majority of patients were on a first trial of NSAIDs. Secondary forms of LöS related to systemic diseases or immune checkpoint blockers, as opposed to idiopathic forms or secondary forms of any other cause, seem to have more aggressive behavior, a hypothesis also formulated by Kim et al. [27]. It is possible that this behavior is related to the severity of the underlying disease per se. However, it is to be considered that the primary condition, especially in the case of systemic diseases, may create some immunomodulation in acute sarcoidosis and influence its progression. The mechanisms underlying this possible interaction are unknown.

A review of case series is presented in Table 3 with the description of 1,276 patients who presented with LöS.

**Table 3:** A list of case series. AA = ankle arthritis; BHL = bilateral hilar lymphadenopathies; EN = erythema nodosum; LöS = Löfgren syndrome

Reference	n	Mean age	M/F	AA or ankle pain	BHL	EN	Follow-up	Treatment	Outcomes
Löfgren (1953) [1,2]	212	25–30	51/161	NS	212 (100%)	113 (53.3%)	Two-year follow-up	NS	Full recovery in the majority of patients (91.9% after two years in the EN group; 72.6% in the non-EN group); 17 out of 62 patients in the non-EN group progressed to chronic sarcoidosis
Caplan et al. (1970) [36]	19	34.0	15/4	17 (89.5%)	19 (100%)	NS	19 patients at one year, and 10 patients at the two-year follow-up	Symptomatic in most patients; one patient received corticosteroid for respiratory impairment after one year	All but two patients were free of disease after one year; 0 recurrences after a two-year follow-up in 10 patients
Pennec et al. (1982) [37]	16	40.1	8/8	15 (93.8%)	NS	11 (68.8%)	15 patients but with variable times of follow-up	NS (majority received symptomatic treatment; only four received steroids)	11 patients fully recovered in less than four years; four patients (25%) had a recurrence or persistent symptoms
Hedfors et al. (1983) [38]	19	33.0	7/12	19 (100%)	19 (100%)	7 (36.8%)	19	NS	All patients fully recovered from AA and EN in three months. Two patients maintained BHL after six months and one year of follow-up, respectively
Valeyre et al. (1984) [39]	14	32.0	0/14	NS	14 (100%)	14 (100%)	14 patients with times of follow-up ranging from 3–12 months	None received steroids	NS
Glennås et al. (1995) [40]	17	30.0	11/6	17 (100%)	17 (100%)	10 (59%)	12 patients after 104 weeks	76% received NSAIDs; 18% had oral corticosteroids	Full recovery of joint pain and EN after 104 weeks
Gran et al. (1996) [41]	49	36.3	30/19	49 (100%)	49 (100%)	43 (87.8%)		The majority was treated with NSAIDs; steroids were introduced to a small proportion of patients with lung parenchymal disease, or to four cases of articular involvement	
Mãná et al. (1996) [42]	33	33.0	11/22	18 (54.5%)	33 (100%)	12 (36.3%)	24	NS	All patients had the inactive disease after one year of follow-up
Wilsher (1998) [43]	59	30.0	27/32	NS	NS	21 (35.6%)	NS	18 patients required steroids	
Yanardag et al. (2003) [44]	98	41.7	17/81	NS	96 (98%)	71 (72.4%)	NS	NS	NS
Demirkok et al. (2006) [45]	87	NS	21/66	31 (35.6%)	63 (72.4%)	87 (100%)	NS	NS	Remission occurred in >50% of patients within the first two years; 2% had recurrence within five years
Thelier et al. (2008) [46]	43	41.0	8/35	39 (91.0%)	39 (91.0%)	39 (91.0%)	NS	88% of patients were treated with NSAID; 16% glucocorticoids; 19% colchicine; and 4% hydroxychloroquine	NS
Grunewald et al. (2009) [47]	301	36.0	165/136	126 (43%)	170 (57%)	301 (100%)	275 for a period of two years	19% required steroids at any point	After two years of follow-up, 81% had a resolving disease, whereas 16% had a non-resolving disease; 3% had a relapsing disease
Rubio-Rivas et al. (2020) [12]	309	39.8	60/249	NS (15.5% of patients had isolated periarticular ankle inflammation)	NS	261 (84.5%)	NS	18.8% were treated, most of them with glucocorticoids	66 patients (21.4%) developed chronic LöS; 25 recurred (8.1%); and 12 relapsed (3.9%)

As previously discussed, a higher proportion of young females was found. Bilateral hilar lymphadenopathies were more frequently described than erythema nodosum, ankle arthralgias, or periarthritis. The majority of patients underwent spontaneous remission or fully recovered after a course of NSAIDs. More than 90% of patients were in remission in less than one year. The description of recurrence, relapse, or chronic sarcoidosis was inconsistent in the series. We found a recurrence rate of 0-25%, a relapse in about 3.9% of cases, and an overall progression to chronic sarcoidosis in 0-27.4% of cases.

## Conclusions

This case report and literature review emphasize the wide web of mimicry of acute sarcoidosis. Despite being associated with an overall good prognosis, the physiopathology of LöS is still widely debated, and the intricacies of its treatment lack further discussion and investigation. From our perspective, secondary forms of acute sarcoidosis are likely to benefit from additional and more complex immunomodulatory therapies. Attention should be paid to systemic diseases that may predispose to LöS, such as autoimmune or neoplastic diseases. Close monitoring and follow-up should also be performed because it is possible that these patients experience higher rates of recurrence or relapse.
